# Pediatric validation of an AI-assisted smartphone application (WebCeph) for cephalometric analysis: reliability, reproducibility and time-efficiency assessment

**DOI:** 10.1590/2177-6709.31.2.e2625274.oar

**Published:** 2026-05-18

**Authors:** Rohan SHINDE, Dinesh RAO, Sunil PANWAR, Manan PHALKE

**Affiliations:** 1 Pacific Dental College & Hospital, Department of Pediatric Dentistry (Udaipur, Rajasthan State, India).

**Keywords:** Cephalometry, Mobile applications, Reproducibility of result, Pediatric dentistry, Cefalometria, Aplicativos móveis, Reprodutibilidade dos resultados, Odontopediatria

## Abstract

**Introduction:**

Although AI-assisted cephalometric applications are widely used, most validation studies involve adult radiographs. Pediatric craniofacial landmarks show greater variability due to growth, which may influence automated landmark accuracy. Therefore, pediatric-specific validation of AI-based smartphone cephalometry is essential.

**Objective:**

This study evaluated the reliability, reproducibility, and time-efficiency of an AI-assisted smartphone application (WebCeph) compared with manual cephalometric tracing in children.

**Methods:**

A retrospective cross-sectional study was conducted on 51 lateral cephalograms of children aged 7-12 years. Manual tracings were performed by a calibrated examiner, and digital tracings were obtained using the WebCeph Android application. Linear and angular measurements were recorded for both methods. Intra-examiner reliability was assessed using intraclass correlation coefficients (ICC). Agreement between methods was evaluated using paired t-tests and Cohen’s d. Tracing time was documented, and normality was confirmed using the Shapiro-Wilk test.

**Results:**

Both methods demonstrated high reliability. Most parameters showed no significant differences, although a few (SNA, ANB, L1-MP) showed statistically significant but clinically negligible discrepancies. ICC values indicated excellent agreement. WebCeph significantly reduced analysis time (9.4 ± 1.82 minutes), compared with manual tracing (19.4 ± 2.65 minutes).

**Conclusion:**

The WebCeph AI-assisted smartphone application provides reliable and clinically comparable cephalometric measurements to manual tracing in pediatric patients, with substantial time savings. It may serve as an efficient adjunct to routine pediatric orthodontic diagnostics, with consideration for growth-related landmark variability.

## RESUMO

**Introdução:**

Embora os aplicativos cefalométricos assistidos por IA sejam amplamente utilizados, a maioria dos estudos de validação usou radiografias de adultos. Os pontos cefalométricos apresentam maior variabilidade em crianças, devido ao crescimento, o que pode influenciar na identificação automatizada dos pontos. Portanto, é essencial validar a análise cefalometria em *smartphone* usando IA especificamente para pacientes pediátricos.

**Objetivo:**

Este estudo avaliou a confiabilidade, reprodutibilidade e rapidez de um aplicativo de *smartphone* assistido por IA (WebCeph), em comparação com o traçado cefalométrico manual em crianças.

**Métodos:**

Um estudo transversal retrospectivo foi realizado em 51 telerradiografias laterais de crianças com 7 a 12 anos. Os traçados manuais foram realizados por um examinador calibrado e os traçados digitais foram obtidos por meio do aplicativo WebCeph para Android. Medidas lineares e angulares foram registradas para ambos os métodos. A confiabilidade intraexaminador foi avaliada por meio de coeficientes de correlação intraclasse (ICC). A concordância entre os métodos foi avaliada por testes t pareados e d de Cohen. O tempo de traçado foi documentado e a normalidade foi confirmada pelo teste de Shapiro-Wilk.

**Resultados:**

Ambos os métodos demonstraram alta confiabilidade. A maioria dos parâmetros não apresentou diferenças significativas, embora alguns (SNA, ANB, L1-MP) tenham mostrado discrepâncias estatisticamente significativas; porém, clinicamente desprezíveis. Os valores de ICC indicaram excelente concordância. O WebCeph reduziu significativamente o tempo de análise (9,4 ± 1,82 minutos), em comparação ao traçado manual (19,4 ± 2,65 minutos).

**Conclusão:**

O aplicativo de *smartphone* assistido por IA WebCeph fornece medições cefalométricas confiáveis e clinicamente comparáveis ao traçado manual, em pacientes pediátricos, com economia substancial de tempo. Pode servir como um adjuvante eficiente para o diagnóstico ortodôntico pediátrico de rotina, considerando-se a variabilidade dos pontos cefalométricos por causa do crescimento.

## INTRODUCTION

The discovery of X-rays by Roentgen in 1895 revolutionized dental radiography.^1^ In 1900, W. A. Price first demonstrated their diagnostic value in Orthodontics, and in 1931, Broadbent and Hofrath introduced the cephalostat, enabling standardized cephalometric techniques.^1^ ^,^ ^2^

Cephalometric radiography remains essential in orthodontic diagnosis, treatment planning, and evaluating craniofacial growth patterns.^3^ ^,^ ^4^ Traditionally, cephalometric analysis was carried out manually using acetate sheets to mark landmarks and measure angles or linear parameters.^3^ ^-^ ^5^ However, manual tracing is time-consuming and prone to operator errors in landmark identification, measurement, and interpretation.^5^ ^-^ ^7^ Moreover, the conversion of three-dimensional structures into two-dimensional images introduces additional inaccuracies.^7^

The advent of digital technology has transformed cephalometric analysis. Ricketts introduced computerized tracing, and ‘Dolphin Imaging’ became a landmark innovation in 1994.^4^ ^,^ ^8^ Digital methods involve digitizing conventional films or using direct digital radiography, offering easier storage, improved image manipulation, and superimposition.^9^ ^-^ ^11^ This method also reduces radiation exposure and eliminates the use of harmful processing chemicals.^12^ Nevertheless, challenges remain, including difficulty in landmark identification and dependence on high-quality digital equipment.^13^

Despite these advances, many practices still rely on digitized conventional films, and the accuracy of smartphone-based cephalometric measurements remains insufficiently explored.^11^ ^,^ ^14^

With their popularity and widespread adoption, smartphones have emerged as powerful tools in healthcare.^15^ ^,^ ^16^ Beyond communication, they function as cameras, navigation systems, and data assistants,^17^ which has encouraged the development of dental applications for diagnosis and patient engagement.^18^ Applications such as OneCeph, CephNinja Pro, Easy Ceph, and WebCeph now allow cephalometric analysis on smartphones.^18^ ^,^ ^19^ These provide rapid analysis and data sharing, with many being freely accessible. Although artificial intelligence (AI) has enhanced the capabilities of digital cephalometric analysis, evidence regarding its clinical accuracy remains mixed.^20^ ^,^ ^21^ While recent studies report promising AI performance, further validation is required before routine clinical adoption.^22^ ^-^ ^24^ WebCeph (AssembleCircle Corp., Republic of Korea), an AI-powered platform, offers automated tracing, treatment simulations, and manual landmark adjustment.^25^ However, conclusive evidence regarding the reliability of these applications remains limited.

Most existing WebCeph validation studies have focused on adult populations, where skeletal landmarks are more stable and well-defined. In contrast, pediatric craniofacial landmarks are highly variable due to ongoing growth, remodeling, and incomplete bone maturation. These age-related differences may influence AI-based landmark detection, creating a need for dedicated validation in children. Therefore, assessing the accuracy of smartphone-based AI cephalometry specifically in pediatric patients is essential for safe and reliable clinical use.

This study addresses the gap in pediatric cephalometric validation by comparing manual tracing with an AI-assisted smartphone application (WebCeph).

## MATERIAL AND METHODS

### STUDY DESIGN

A retrospective, cross-sectional, *in vitro* study was conducted in the Department of Pediatric and Preventive Dentistry, to evaluate and compare the reliability and reproducibility of linear and angular lateral cephalometric measurements from an Android-based application (WebCeph) with manual tracing in children aged 9-15 years. Ethical approval for this study (Ref No. STU/IEC/2023/251) was provided by the Ethical Committee Sai Tirupati University, Udaipur, on 21 October 2023. This was a retrospective *in vitro* investigation utilizing archived radiographs without direct patient involvement; thus, the committee waived the necessity for informed consent.

### SAMPLE SIZE AND SELECTION CRITERIA

The sample size for the study was guided by samples taken from similar previous studies.^5^ The sample size was calculated using the software G^*^power (version 3.1.9.7, Germany) that calculates sample size based on the following formula:

$$[N\;=\;{\left(Z1-\alpha /2\text{​}+Z1-\beta \text{​}\right)}^{2}\left(k-1\right)\left(1+\left(k-1\right)\rho 0\text{​}\right)\text{​}/\;k{\left(\rho 1\text{​}-\rho 0\text{​}\right)}^{2}.$$

Using test power (0.8) with error buffer (10%) for calculating effect size at a Simple α level of 0.05 and 95% confidence interval, the sample size was estimated to be 51 similar radiographic analysis for each group. 

### OPERATIONAL DEFINITIONS

Pixels per inch (PPI) indicate the pixel density of radiograph on a monitor, whereas dots per inch (DPI) reflects printer resolution, with higher values yielding sharper prints. A platform refers to the computing environment supporting the software. In cephalometric radiography, standardization ensures that image size and resolution correspond to a true 1:1 representation of anatomical structures. 

### SAMPLE SELECTION

The cross-sectional study utilized a randomly selected sample of 51 lateral cephalograms retrieved from archives. Lateral cephalometric radiographs of medically fit individuals aged 9-15 years with fully erupted permanent first molars, no evident craniofacial deformity or asymmetry, and taken in maximal intercuspation under standardized conditions using the same digital device and magnification, were included. Only images of high quality and free from artifacts were considered eligible. Radiographs were excluded if anatomical landmarks could not be clearly identified due to motion blur, poor resolution, or contrast deficiency, or if gross craniofacial deformities, artefacts, or pathological lesions were present. All radiographs were retrieved from institutional archives within the preceding 2 years by an orthodontist not involved in the study and blinded to the study objectives. 

### DIGITAL CALIBRATION

All radiographs were digitally calibrated using dots-per-inch (DPI) or pixels-per-inch (PPI) values from the scanner device, eliminating the need for high-resolution printers and allowing compatibility up to 300 DPI.^13^ Each image was adjusted to match actual anatomical dimensions, and hard copies were printed using the same software to prevent distortion. Radiographs were saved in JPEG format and printed.

For calibration in the smartphone application, the inbuilt calibration ruler was used to define two points on each image, ensuring accurate scaling to real-world dimensions. All devices were calibrated and corrected for magnification with the millimeter calibration ruler, to maintain measurement accuracy.

### RADIOGRAPHIC TRACING

All tracings were carried out by a single operator, blinded to the study objectives and outcome measurements, to eliminate inter-examiner variability. The operator was a postgraduate student with two years of clinical experience, and was trained in the number of radiographs to be processed per day and the extent of analysis required. Accurate landmark identification was supervised by a senior professor with ten years of specialty experience. To minimize fatigue-related errors, no more than five tracings were performed per working day.

### MANUAL CEPHALOMETRIC TRACING

**Figure 1:**
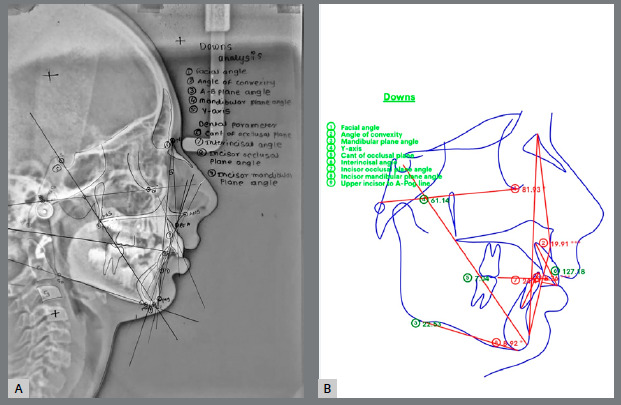
Down’s analysis: **A**) manual tracing, **B)**WebCeph application.

Lateral cephalometric images were printed at a 1:1 scale and traced in the morning hours on an illuminated view box, in a dark room. A single examiner performed all cephalometric tracings. Acetate tracing paper was fixed over each radiograph, with two reference crosses (~3 cm apart) marked for superimposition and patient details recorded. Hard tissue outlines including cranial base, upper and lower central incisors, first molars, maxilla, and mandible were traced, identifying and tracing the landmarks: S, N, Po, Or, Pt, Cd, Ar, PNS, ANS, A, B, Pog, Go, Gn, and Me. The landmarks and reference points for Steiner’s, Down’s, Tweed’s, and Jarabak’s analyses were identified and marked, and corresponding angular and linear measurements were recorded (Fig. 1).

### ANDROID CEPHALOMETRIC TRACING

The WebCeph application was installed from the Google Play Store on a compatible Android smartphone. An account was created within the application, for data management. The smartphone featured a 6.40-in AMOLED display with a resolution of 1080 × 2400 pixels, which was used for viewing parameters and analyzing the cephalograms. Patient details and radiographs (uploaded via camera or storage) were added to individual records. During analysis, the distance between the smartphone and the viewing surface was maintained at approximately 6-in.

**Figure 2:**
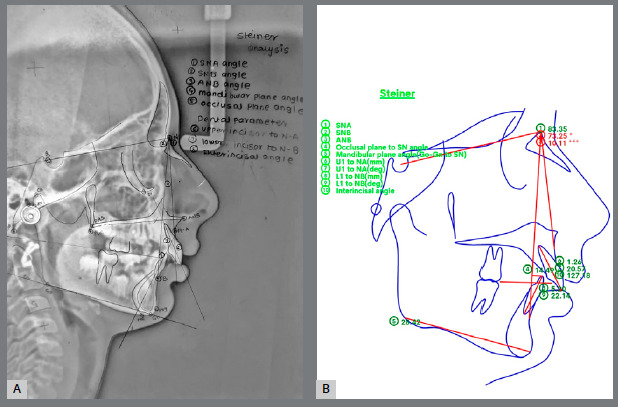
Steiner’s analysis: **A**) manual tracing. **B**) WebCeph application.

The “A.I. Digitalisation” tool was employed for automatic landmark identification, with options for calibration, modification, or saving as required. Selected analyses automatically generated angular and linear measurements, which were saved and exported as PDF reports. The time taken for each tracing was recorded for statistical analysis. Landmarks and reference points for Steiner’s, Down’s, Tweed’s, and Jarabak’s analyses were identified and marked, and the corresponding angular and linear measurements were documented (Fig. 2).

Furthermore, 15 randomly selected radiographs were retraced and remeasured by the same examiner at a 7-day interval, after the initial assessment, to evaluate the magnitude of intra-examiner variability. The estimated random error was assessed utilizing Dahlberg’s test.

### STATISTICAL ANALYSIS

Data were analyzed using IBM SPSS statistics 28.0 (IBM Corp., Armonk, NY, USA). Paired t-tests were used to compare manual and Android-based smartphone application measurements. Effect size was calculated using Cohen’s d. Intra-examiner reliability was evaluated with intraclass correlation coefficients (ICC). A significance level of p < 0.05 was adopted. Difference in tracing time and cephalometric measurements were assessed for statistical significance. Data distribution was assessed using the Shapiro-Wilk test, confirming normality prior to applying parametric tests.

## RESULTS

Intra-observer reliability analysis using Dahlberg’s formula revealed an overall method error of 1.90. In addition, the ICC, calculated using a two-way mixed model with absolute agreement, demonstrated good reproducibility of landmark identification over time by the same examiner (Table 1).

**Table 1:** Intra-observer error analysis of cephalometric measurements.

Analysis	Parameter type	Manual tracing (ICC)	WebCeph (ICC)
Downs	Angular & linear	0.960 - 0.993	0.868 - 0.993
Steiner	Angular & linear	0.949 - 0.994	0.866 - 0.998
Tweed	Angular	0.961 - 0.986	0.881 - 0.986
Jarabak	Angular & linear	0.949 - 0.994	0.897 - 0.994

ICC: intraclass correlation coefficient. < 0.5 = poor; 0.5 - 0.75 = moderate; > 0.90 = excellent agreement.

Down’s analysis showed no significant differences between the two methods for most parameters, except for the cant of the occlusal plane (*p*= 0.041) and L1-MP (*p*= 0.001), which demonstrated statistically significant differences (Table 2).

**Table 2:** Downs’ parameters: Manual vs WebCeph.

Parameter	Manual (Mean ± SD)	WebCeph (Mean ± SD)	t value	p value	Cohen’s d
Facial angle (degrees)	85.30 ± 2.65	85.58 ± 2.84	-1.366	0.188	-0.10
Angle of convexity (degrees)	8.11 ± 4.93	9.47 ± 6.09	-1.950	0.066	0.25
Mandibular plane angle (degrees)	24.05 ± 4.18	23.93 ± 4.41	0.359	0.724	0.03
Y-axis angle (degrees)	59.75 ± 1.71	59.50 ± 2.20	0.511	0.615	0.13
Cant of occlusal plane (degrees)	9.52 ± 5.54	7.74 ± 5.69	2.093	0.041*	0.32
Interincisal angle (degrees)	128.05 ± 10.96	127.77 ± 10.88	0.590	0.562	0.03
L1.OP (degrees)	29.60 ± 16.76	24.87 ± 6.98	1.522	0.145	0.37
L1.MP (degrees)	32.85 ± 27.64	13.91 ± 15.37	3.925	0.001**	0.85
U1-A-Pog (mm)	7.32 ± 3.77	7.65 ± 4.14	-1.244	0.228	-0.08

NS = not significant; *p < 0.05 = significant; **p < 0.01 = highly significant.

Cohen’s interpretation: negligible (<0.2), small (0.2-0.9), medium (0.5-0.79), large (≥0.8).

Steiner’s analysis revealed significant differences in SNA (*p*= 0.042), ANB (*p*= 0.034), U1.NA (degrees) (*p*= 0.034), U1-NA (mm) (*p*= 0.018), and L1-NB (mm) (*p*= 0.023), while other parameters were comparable between the two methods (Table 3).

**Table 3:** Steiner’s parameters: Manual vs WebCeph.

Parameter	Manual (Mean ± SD)	WebCeph (Mean ± SD)	t value	p value	Cohen’s d
SNA (degrees)	81.55 ± 2.09	80.95 ± 2.43	-2.186	0.042*	0.26
SNB (degrees)	76.17 ± 3.42	76.22 ± 3.47	0.185	0.855	0.01
ANB (degrees)	5.25 ± 2.92	4.59 ± 2.22	-2.289	0.034*	0.25
Occlusal plane-SN (degrees)	14.49 ± 6.19	15.32 ± 5.69	1.878	0.076	0.14
Mandibular plane-SN (degrees)	29.85 ± 4.66	30.00 ± 4.41	0.448	0.659	0.03
U1.NA (degrees)	23.05 ± 8.78	22.55 ± 8.42	-2.286	0.034*	0.06
U1-NA (mm)	3.96 ± 2.31	3.73 ± 2.02	-2.595	0.018*	0.10
L1.NB (degrees)	23.85 ± 4.61	23.80 ± 5.03	-0.116	0.909	0.01
L1-NB (mm)	5.38 ± 2.29	4.91 ± 2.61	-2.465	0.023*	0.19
Interincisal angle (degrees)	126 ± 8.84	125.90 ± 8.97	0.717	0.482	0.13

NS = not significant; *p < 0.05 = significant; **p < 0.01 = highly significant

Cohen’s interpretation - negligible (<0.2), small (0.2-0.9), medium (0.5-0.79), large (≥0.8).

Tweed’s analysis indicated that only the Frankfort-mandibular plane angle (FMPA) showed a statistically significant difference (*p*= 0.011), whereas IMPA and FMIA values were not significantly different (Table 4).

**Table 4:** Tweed’s parameters: Manual vs WebCeph.

Parameter	Manual (Mean ± SD)	WebCeph (Mean ± SD)	t value	p value	Significance
FMPA (degrees)	23.71 ± 4.18	24.40 ± 3.87	2.806	0.011*	-0.17
IMPA (degrees)	93.99 ± 10.84	94.75 ± 13.63	0.416	0.682	-0.06
FMIA (degrees)	62.85 ± 11.07	62.71 ± 10.08	-0.344	0.735	0.01

NS = not significant; *p < 0.05 = significant; **p < 0.01 = highly significant.

Cohen’s interpretation: negligible (< 0.2), small (0.2-0.9), medium (0.5-0.79), large (≥ 0.8).

Jarabak’s analysis demonstrated no significant differences for angular measurements, while among linear parameters, anterior cranial base (*p*= 0.043) and posterior cranial base (*p*= 0.033) showed significant differences between methods. Other linear and angular variables did not differ significantly (Table 5).

**Table 5:** Jarabak’s parameters: Manual vs WebCeph.

Parameter	Manual (Mean ± SD)	WebCeph (Mean ± SD)	t value	p value	Cohen’s d
Saddle angle (degrees)	122.51 ± 5.74	122.45 ± 5.61	-0.298	0.769	0.01
Articular angle (degrees)	151.00 ± 7.51	150.10 ± 7.51	-1.044	0.309	0.12
Gonial angle (degrees)	121.05 ± 5.81	121.45 ± 3.53	0.524	0.606	-0.08
Jarabak sum (degrees)	391.02 ± 18.24	394.05 ± 7.63	0.824	0.420	-0.22
Upper gonial angle (degrees)	50.07 ± 4.39	50.65 ± 4.34	1.454	0.162	-0.13
Lower gonial angle (degrees)	70.16 ± 4.22	70.65 ± 3.64	1.874	0.076	-0.12
Anterior cranial base (mm)	66.80 ± 3.13	66.30 ± 3.37	-2.169	0.043*	0.15
Posterior cranial base (mm)	29.29 ± 3.87	28.50 ± 4.68	-2.300	0.033*	0.18
Ramus height (mm)	41.96 ± 4.06	41.55 ± 4.14	-2.016	0.058	0.10
Anterior facial height (mm)	107.04 ± 8.11	106.62 ± 8.09	-1.015	0.323	0.05
Posterior facial height (mm)	68.58 ± 6.56	69.05 ± 7.06	1.456	0.162	-0.07

NS = not significant; *p < 0.05 = significant; **p < 0.01 = highly significant.

Cohen’s interpretation: negligible (< 0.2), small (0.2-0.9), medium (0.5-0.79), large (≥ 0.8).

Time efficiency analysis showed that android smartphone application (WebCeph) significantly reduced the time required for tracing (mean 9.41 ± 1.82 minutes), compared to manual tracing (mean 19.41 ± 2.65 minutes), with a highly significant difference (*p*< 0.001) (Table 6).

**Table 6:** Time efficiency: Manual vs WebCeph.

Method	Mean ± SD (min.)
Manual tracing	19.41 ± 2.65
WebCeph	9.41 ± 1.82

## DISCUSSION

Cephalograms remain indispensable in Orthodontics and Pediatric Dentistry for diagnosing skeletal and dental discrepancies and evaluating treatment outcomes.^6^ ^-^ ^7^ With technological advances, manual tracing is increasingly replaced by digital systems and smartphone-based applications, offering advantages such as reduced radiation, improved storage, and image manipulation.^25^ However, any method must be reliable, precise, and reproducible.^26^ This study compared the reliability and reproducibility of an Android-based application (WebCeph) with the ‘gold standard’ manual tracing among children aged 9-15 years. WebCeph, FDA- and KFAD-approved, uses AI trained on craniofacial datasets^27^, but its smartphone performance required validation. 

A relatively large sample (n=51) was used, and all tracings were performed by a single examiner, to avoid inter-observer bias, as landmark identification accuracy strongly influences analysis.^5^ ^,^ ^28^ Intra-observer reliability analysis using Dahlberg’s formula revealed an overall method error of 1.90. This value lies within the clinically acceptable range for angular measurements (≤2º) and was slightly higher than the ideal threshold for linear measurements (≤1mm). Intra-examiner reliability showed minimal differences between repeated tracings, consistent with previous studies.^28^ Out of 31 measured parameters from Down’s, Steiner’s, Tweed’s, and Jarabak’s analyses, 21 showed no significant difference between methods, while 10 exhibited statistically significant but clinically insignificant variations. Statistically significant differences in parameters such as SNA, ANB, and L1-MP may be attributed to AI landmark detection sensitivity in areas where anatomical boundaries are less distinct in growing children. Growth-related remodeling of the maxilla and mandible can alter landmark clarity, and algorithmic smoothing may influence angular estimation.^13^ ^,^ ^27^ Despite these differences, most measurements fell within acceptable norms, indicating that WebCeph is reliable for clinical use, with caution in interpreting specific parameters.^29^ ^,^ ^30^

The application significantly reduced analysis time (19.4 ± 2.65 minutes vs. 9.4 ± 1.82 minutes), corroborating previous findings.^5^ ^,^ ^27^ Additional advantages include free access, multilingual support, compatibility across devices, image calibration, manual correction, and customizable analyses. Given its speed, accessibility, and high reliability for most parameters, the Android-based application (WebCeph) is a viable tool for routine orthodontic diagnosis, though further studies with larger samples are recommended to confirm its reproducibility. Since WebCeph is cloud-based, secure handling of patient radiographs is essential. The platform uses encrypted data transfer, but clinicians must ensure compliance with institutional and national data-protection protocols.

The study is limited by its single-operator design, which prevents evaluation of inter-examiner variability. The sample was geographically homogeneous, and results may differ across populations. The analysis was restricted to 2D lateral cephalograms; comparison with 3D CBCT-based landmarks could provide further insight. Although the WebCeph algorithm is trained on large datasets, most AI datasets are predominantly adult-based. Pediatric craniofacial structures undergo continuous growth-related changes, which may reduce landmark identification accuracy and contribute to the minor discrepancies observed in parameters such as SNA, ANB, and L1-MP.

Within the limitations of this study, the Android-based application (WebCeph) showed reliability and reproducibility comparable with manual tracing. The observed statistically significant differences were clinically negligible, while analysis time was significantly reduced. Given its accessibility and ease of use, WebCeph offers a practical alternative for routine cephalometric analysis in pediatric and orthodontic practice. Further multicentric studies are necessary to validate its applicability across many populations and clinical settings.

## CONCLUSIONS

1. This study demonstrates that a smartphone-based application (WebCeph) provides reliable and reproducible cephalometric analysis in children, comparable to manual methods.
2. The application reduces analysis time by more than 50%, while maintaining clinically acceptable accuracy.
3. These findings support the use of accessible, cost-effective digital tools in pediatric dental practice.
4. Integration of such technology can improve diagnostic efficiency, patient communication, and clinical workflow.

## Data Availability

Due to the sensitive nature of the data and ethical restrictions, the datasets supporting this study are not publicly available.
